# Brain MR Image Classification for Alzheimer's Disease Diagnosis Based on Multifeature Fusion

**DOI:** 10.1155/2017/1952373

**Published:** 2017-05-22

**Authors:** Zhe Xiao, Yi Ding, Tian Lan, Cong Zhang, Chuanji Luo, Zhiguang Qin

**Affiliations:** ^1^School of Information and Software Engineering, University of Electronic Science and Technology of China, Chengdu, Sichuan Province 610054, China; ^2^China Gas Turbine Establishment, Mianyang, Sichuan 621000, China

## Abstract

We propose a novel classification framework to precisely identify individuals with Alzheimer's disease (AD) or mild cognitive impairment (MCI) from normal controls (NC). The proposed method combines three different features from structural MR images: gray-matter volume, gray-level cooccurrence matrix, and Gabor feature. These features can obtain both the 2D and 3D information of brains, and the experimental results show that a better performance can be achieved through the multifeature fusion. We also analyze the multifeatures combination correlation technologies and improve the SVM-RFE algorithm through the covariance method. The results of comparison experiments on public Alzheimer's Disease Neuroimaging Initiative (ADNI) database demonstrate the effectiveness of the proposed method. Besides, it also indicates that multifeatures combination is better than the single-feature method. The proposed features selection algorithm could effectively extract the optimal features subset in order to improve the classification performance.

## 1. Introduction 

In recent years, Alzheimer's disease has become a common neurodegenerative brain disease in elderly people. According to a report published by Alzheimer's Disease International, there are around 44 million dementia patients worldwide, and the number will reach 76 million by 2030 and 135 million by 2050. Among these patients, Alzheimer's disease (AD) patients account for 50% to 75% [1] characterized by insidious onset and progressive impairment of episodic memory [2]. Mild cognitive impairment (MCI) is a condition in which an individual has mild but noticeable changes in thinking abilities. Individuals with MCI are more likely to develop AD than individuals without it [3]. Although there are no medications to cure AD, some medications have been used to delay the onset of some symptoms and reduce psychological impact on the patients, such as memory loss [4]. Therefore, accurate diagnosis of AD patients or MCI in the early stage is very important.

At present, machine learning and pattern classification methods have been widely utilized in developing a computer-aided brain disease diagnosis system with neuroimages such as Magnetic Resonance Imaging (MRI) [5], Positron Emission Tomography (PET) [6], functional MRI (fMRI) [7], and Diffusion Tensor Imaging (DTI) [8]. Studies have shown that structural MRI is the most standardized imaging modality in clinical practice [9] and it is also useful to track different clinical phases of AD [10]. Therefore, our method is evaluated on structural MR images.

Several types of features can be extracted from the structural MRI of whole brain, such as intensities or gray-matter densities [11], group comparison of cortical thickness [12], morphometry [13], and texture measures [14]. The combination of different types of features can improve the accuracy of the AD diagnosis in comparison to methods which use just a single feature [15].

Texture analysis could analyze the subtle changes of body; therefore, it has been widely used in AD diagnosis for extracting texture features. Oliveira et al. [16] adopted a statistical method to differentiate cerebral MR images of patients with AD and amnestic mild cognitive impairment (MCI). In the paper proposed by Torabi et al. [17], 336 features extracted from gray-level cooccurrence matrix (GLMC) were used for classification of AD. Ghorbanian et al. [18] used discrete wavelet transform to extract features to diagnose AD. At present, morphometric MRI can be adapted to improve the performance of diagnosing AD, which is safe, reliable, and noninvasive. Analysis based on region-of-interest (ROI) measurements and voxel-based morphometry (VBM) is widely used to evaluate morphometric changes [19]. In particular, VBM is sensitive and hypothesis-free in terms of localizing small-scale regional differences in gray matter [20], so it has been commonly applied to the study of gray-matter alterations in AD [21–23]. We also used the combination of VBM analysis and texture analysis to study AD in this paper.

Moreover, the number of feature dimensions in neuroimaging is commonly higher than the number of samples. In order to solve the problem of overfitting, it is necessary to select features. Common feature selection algorithms can be divided into three categories: filters, wrappers, and embedded approaches. To be more specific, filter methods select subsets of features using learning algorithms, like principal components analysis (PCA) [24]. However, wrapper and embedded methods use learning machine to evaluate subsets of features according to their predictive performance. In the classification based on neuroimaging, several feature selection techniques have been proposed, for example, univariate methods (e.g.,* t*-test) [25], multivariate approaches (e.g., sparse logistic regression) [26], perturbation method [27], and support vector machine recursive feature elimination (SVM-RFE) [28]. SVM-RFE has been successfully implemented in various neuroscience applications [29, 30], but it does not have a good performance on image analysis [31]. In this paper, we improve the process of feature selection by combining the SVM-RFE and covariance. Moreover, we also realized some proposed learning algorithms, like multiple kernel learning algorithms [32]. It shows good performance in Alzheimer's disease diagnosis [33–36]. Because of this, multikernel learning is taken as a comparison method in this paper.

In this paper, we propose a novel classification framework to precisely identify individuals with Alzheimer's disease (AD) or mild cognitive impairment (MCI) from normal controls (NC). Firstly, we propose a combination of voxel-based morphometry (VBM) and texture analysis to extract the more discriminative features. To be more specific, the VBM analysis can obtain the 3-D information of the brain, and texture analysis can obtain the 2-D information, so fusing the two kinds of features can achieve a better performance. Secondly, we adapt the SVM-RFE with covariance method to select a robust feature subset and use it to solve the overfitting problem of feature fusion. Our proposed method is evaluated on Alzheimer's Disease Neuroimaging Initiative (ADNI) database and shows better performance than comparison methods. The following part of the paper is organized as follows: in Section 2, the details of the proposed framework and related methods are described; in Section 3, experimental setting, results, and analysis are presented; in Section 4, we conclude our work and briefly outline the future work.

## 2. Methods

**Figure 1:**
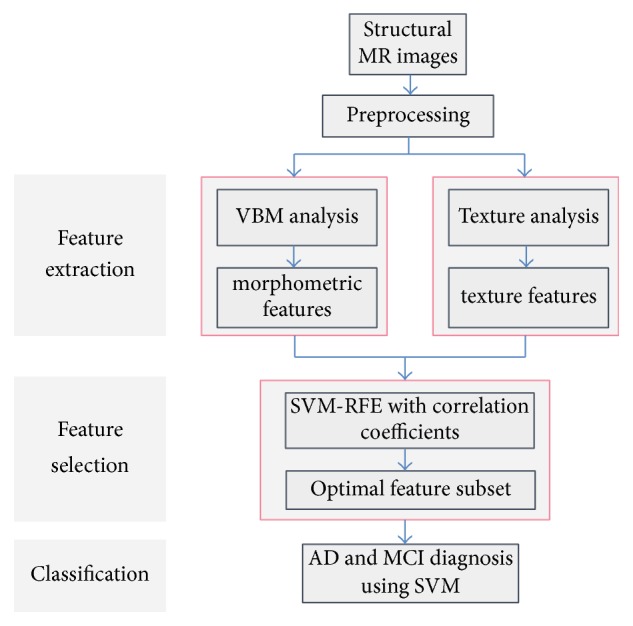
Schematic diagram illustrating the proposed AD and MCI classification framework.

In this section, the proposed framework will be described in detail. An overview of the proposed classification method is illustrated in Figure 1.

### 2.1. Image Preprocessing

We use the ADNI pipeline as introduced in Jack et al. [37] to preprocess the structural MR images. It includes 4 steps as follows: (1) postacquisition correction of gradient warping, (2) B1 nonuniformity correction, (3) intensity nonuniformity correction, and (4) phantom-based scaling correction. After executing these 4 steps, the skull is stripped. The nonbrain tissues are removed by using the method proposed in Leung et al. [38].

### 2.2. Feature Extraction

In this paper, the schematic diagram of the proposed AD and MCI classification framework is shown in Figure 1. Texture features are the most commonly used traditional low-level features, it is because different texture features can reflect the different characteristics of the image, and we take GLCM and Gabor filter to extract texture features. Beyond that, we selected a set of typical images from image volumes under the guidance of clinical doctors in order to analyze the textures accurately. On the other hand, voxel-based morphology has a strong practicality in the field of brain medical image such as brain surgery [39]. Due to the experimental data being structural MR images, we adapt VBM analysis to extract morphometric features.

(*1) VBM Analysis*. VBM is capable of investigating gray-matter abnormalities across the whole brain [40], so the features are extracted from clusters with significant gray-matter differences among AD patients, MCI patients, and NC. Steps for extracting morphometric features using VBM are listed as follows.

**Step 1 (improved VBM spatial normalization).**

Generally speaking, the value of image's density will be changed when the image is processed by the traditional spatial normalization. And these changes will lead to the local error in subsequent brain tissue segmentation, such as the false segmentation of brain tissue or nonbrain tissue affecting the accuracy of analysis. In order to solve this problem, Good et al. [41] proposed an improved VBM method. This improved VBM method can not only improve the segmentation accuracy but also calculate the density information and original volume information of brain tissue, but it did not improve the precision of registration. Therefore, DARTEL (diffeomorphic anatomical registration through exponential lie algebra) [42] was proposed. DARTEL ensures the registration result is diffeomorphic based on the deformation field. Therefore, the improved VBM-DARTEL method not only improves the accuracy of the segmentation and retains the original volume information but also ensures the precision of spatial normalization.

**Step 2 (segmentation of brain tissue).**

After the processing of spatial normalization, the images are segmented into gray matter (GM), white matter (WM), and cerebrospinal fluid (CSF). Because brain tissue segmentation is based on voxel brightness, the different groups segmented using that will be affected by smooth brightness changes and cause a problem of nonuniform brightness, so current brain tissue segmentation technology will include the correction of image nonuniform brightness.

**Step 3 (spatial smoothing).**

Spatial smoothing is a filtering process based on images of different tissue segmented by the last step. Gauss kernel function in normalized space is often used to implement a convolution on image data and the half width and height range of the Gauss function is 4 mm~10 mm. In general, the smoothing process has the ability to eliminate the subtle matching error and to improve the signal-to-noise ratio. However, spatial smoothing has some other effects for VBM. For example, it can make the analysis result based on voxel equal to the result based on ROI (region of interest). Consequently, every voxel of image after spatial smoothing can contain the mean concentration of GM from voxel statistics, which is the so-called “gray-matter density.” Notice that it is far different from the biological cell packing density. According to the central limit theorem, the process of smoothing also has an effect to conform the data to normal distribution. After that, the effectiveness of subsequent statistical analysis can be improved.

**Step 4 (VBM statistical analysis).**

In general circumstances, VBM is often used for the quantitative analysis of the gray matter. Therefore, we adapt the GM images to implement statistical analysis. At present, a commonly used VBM statistical analysis method is based on GLM [43]. This method implements a two-sample* t*-test tactic on hypothesis to check whether the density difference in a region of the two groups of gray image is significant. We can obtain the regional information with a significant density difference in the GM images after FDR correction. According to this information, the clusters can be obtained to make ROI binary masks. Finally, we extract the gray-matter volume from the GM images as morphometric features by applying these masks in REST v1.6 (http://restfmri.net/forum/).

(*2) GLCM*. Gray-level cooccurrence matrix proposed by Haralick et al. [44] is a texture feature extraction method based on gray-level spatial dependence. From these GLCMs, a total number of 28 texture descriptors can be computed. In this paper, we generated 20 gray-level cooccurrence matrices on every brain MRI and 11 features were employed, including angular second moment, sum of squares, inverse difference moment, sum averages, sum variances, contrast, entropy, correlation, difference variance, sum entropy, and difference entropies. 

(*3) Gabor Filters*. Gabor filter performs well in both spatial and frequency domain of any number of dimensions [45]. In this paper, we used two-dimensional Gabor filters with different frequency and direction to extract the texture features. Finally, the mean and variance of the amplitude are calculated as the texture feature values extracted by each filter. The feature value can reflect the concentration trend and discrete distribution of the local energy spectrum of the image.

### 2.3. Feature Selection

After the feature extraction, the linear combination of morphometric features and texture features can be yielded. Unfortunately, these features are less effective, irrelevant, and redundant for classification. Therefore, the feature selection process is essential for selecting an optimal feature subset to improve the classification performance. 

*(1) SVM-RFE*. SVM-RFE is a heuristic-based encapsulation feature selection method based on SVM, which is used to study the gene selection of cancer classification. The SVM-RFE is popular in the field of gene analysis and then gradually applied to neuroscience and medical images and achieved good application results [30].

SVM-RFE is a supervised loop iterative cancellation method. During each iteration, the SVM training process performs first, and the optimal hyperplane is obtained. Then, the ranking score is the weight corresponding to the feature calculated according to the parameters of the hyperplane. Finally, remove the feature with minimum score from the feature sets, and this loop ends. Check whether the feature set at this time only holds one single feature. If there are two or more features, the looping process is continued until only one feature is left. Thus, outputting the sorted result for all the features, it is known from the above process that the feature sequences are arranged in descending order.

*h* represents *h*_th_ feature. During the SVM process, *ω* is the weight vector of feature, so the ranking score *c*_*h*_ is calculated as shown in (1) for the linear case.

(1) ch=ωh2ω=∑i=1nαiyixi,

where *ω*_*h*_ denotes *h*_th_ value and corresponds to the weight of *h*_th_ feature. In the case of nonlinearity, Guyon assumes that the optimal hyperplane does not change even if the feature vectors change; that is, the parameter *α*_*i*_ does not change. Then the corresponding ranking scores are calculated as shown in

(2) ch=12αTHα−12αTH−hαHij=Kxi,xj,

where *H* is a matrix and (−*h*) indicate *h*_th_ eliminated feature.

However, according to Guyon's experiments in the literature, the results are similar in both situations (linear and nonlinear kernels are used). The higher ranking score is, the more contribution the feature makes in classification training. Based on this idea, SVM-RFE finally obtains a descending feature sequence. According to this descending list, we can define a set with *h* features, and *h* is 1 to the total number of features. And then we filtered these subsets by the classification effect with SVM to generate the optimal feature subsets. 

*(2) Covariance*. The covariance matrix provides the correlation analysis between two sets of observed variables. Taking two random variables *X* and *Y* as examples, let *N* be the number of two variables, and then the covariance is calculated as

(3) $$\begin{array}{c} cov\left(\;X,Y\;\right)=\overset{N}{\underset{i=1}{\sum }}\frac{\left(x_{i}-\bar{x}\right)\left(y_{i}-\bar{y}\right)}{N}, \end{array}$$

where $\overset{-}{x}$ and $\overset{-}{y}$ are the mean values of the two sets of variables, respectively.

It can be seen that the covariance matrix is a symmetric matrix, reflecting the observation of the correlation between the two variables; if a positive value represents that the two variables are positive correlation, negative values indicate a negative correlation. It also shows the importance and redundancy of the variable. If the diagonal elements are small, it indicates that this variable is likely to be a secondary variable, and the nondiagonal element value corresponds to the redundancy degree between the variables. 

**Figure 2:**
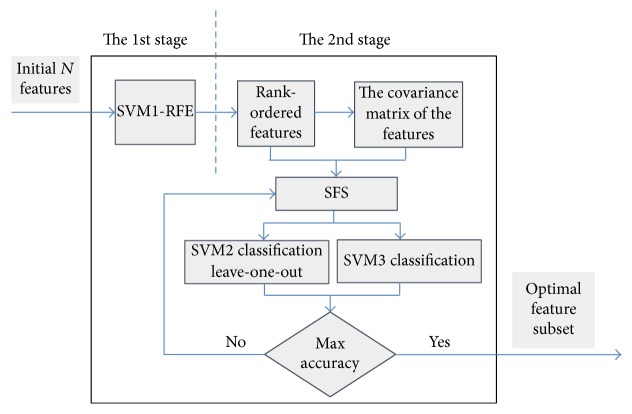
The SVM-RFE with covariance scheme.

*(3) SVM-RFE with Covariance*. In this paper, the SVM-RFE is combined with covariance matrix to optimize selection process of feature subset in order to obtain a robust optimal feature subset. If the SVM-RFE is executed alone, it may not yield optimal feature subset. As illustrated in Figure 2, the improved method can be divided into two stages. At the first stage, the SVM-RFE is implemented with the selected parameters of SVM classifier which are obtained from the suggestion of Guyon in the literature or through training all sample data. At the second stage, covariance matrix of all feature vectors is calculated at the beginning and then combined with the feature sorting result from the first stage. At the next step, we employed the sequential forward selection (SFS) method to generate feature subsets. Finally, we use SVM classification verification testing to generate the optimal feature subset.

To be more specific, the initial state of the subset is empty and SFS process's function is to select iteratively one feature to add to it. The selected feature is the highest ranked feature among unselected features of the ordered feature set or it is related to the highest ranked feature according to the covariance matrix of the features. In other words, in the process of SFS, a parameter *K* is set to control how much features are related to the selected feature with highest ranking. Meanwhile, in order to test the stability of features selected by SFS and verify the effectiveness of this method, the SVM training and testing will be implemented together on that feature subset in each iteration, and the training process takes leave-one-out method for cross validation to ensure the unbiasedness of the result. Next, divide all samples into two sample sets in a ratio of 3 : 1. Keep the parameters of SVM2 and SVM1 the same, but SVM3 uses the optimized parameters obtained by grid search method. Finally, through comparing the classification accuracy of two sample sets, we can get the optimal feature subset when they show a high accuracy at the same time.

### 2.4. Methods Setting

Several parameters are needed to be defined in the proposed method. In the texture feature extraction stage, gray-level cooccurrence matrix is used to extract the features, the selected spatial distance is from 1 to 5 pixels, and directions are 0°, 45°, 90°, and 135°. A total of 20 gray-level cooccurrence matrices are constructed, and then there are 11 quadratic statistics including contrast values that are extracted from the matrix; Gabor filter's extracting window size is 3 × 3, and the selected frequencies and directions are 0.5, 0.25, 0.125, and 0.1 and 0°, 45°, 90°, 180°, and 225°, 315°. A total of 32 filters were constructed, and the gray-level mean and gray-level variance are calculated from each filter response. At the stage of morphological feature extraction, the spatial normalization process is VBM-DARTEL, and the image is registered to the MNI space. The 8 mm FWHM Gauss kernel function is used to smooth the image, and then the GM images are used for statistical analysis.

The feature selection is implemented after the feature normalization. We let *K* represent the number of features that are related to the selected highest ranked feature. It can be proven that when selecting the relative feature by employing the covariance, *K* features, which are selected beginning from the minimum covariance, are more effective than *K* features selected from the maximum covariance. The parameters in SVM_3_ were searched by grid search, and the parameters in SVM_2_ are the same as in SVM_1_.

The proposed method has been compared with five other methods in order to evaluate the effectiveness. Due to the limited number of samples, during the experiment, leave-one-out method is used to search for the optimal kernel function parameter pair, and then 10-fold cross validation is used to classify the sample data. Three random assignments were performed to guarantee random sample partitioning without sample bias. That is to say, every experiment in this article is randomly divided into three samples; in each time, the implementation of 10-fold cross validation ensures the fairness of the results. Moreover, the grid search was used to optimize the parameter for each model. In the experiment, the SPM and REST were implemented for extracting the morphological features, and a shogun toolbox (http://www.shogun-toolbox.org/) was adopted for the SVM classifier learning.

## 3. Experiments and Results

### 3.1. Materials

We obtained all data in our experiments from ADNI public database. The sample images were using 1.5 T scanner and T1 weighted MRI. A total number of 170 subjects were enrolled, including 54 patients with Alzheimer's disease (AD), 58 mild cognitive impairments (MCI), and 58 normal controls (NC). The demographic statistics of these samples are shown in Table 1. The proportion of men and women in AD, MCI, and NC, age information, and Mini Mental State Examination (MMSE) are listed in the table. The evaluation criteria for MEMS are as follows: (1) MMSE results range from 24 to 30, and no other known diseases, such as depression, can be considered the normal control; (2) if MMSE results are from 20 to 26, at the same time in line with the relevant standards of AD issued by national research institutions, then they may be AD patients.

According to the statistical information in the table, the sex ratio of AD, MCI, and NC is balanced. The mean age of three groups is about 75 years, and the MMSE of AD is lower than MCI, which is lower than NC.

### 3.2. Group Differences in Gray-Matter Volume

**Figure 3:**
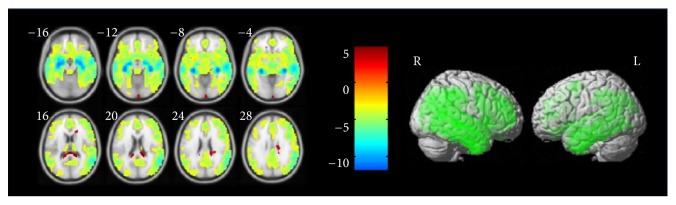
Significant GM difference in AD relative to NC.

(*1) AD-NC (Alzheimer's Disease Comparing with Normal Controls).* After comparing the two-sample* t*-test of the normal controls and Alzheimer's disease group maps, we can get information of the significant clusters of activation, as shown in Table 2, which employs an uncorrected threshold with the value of *P*_uncorrected_ ≤ 0.001, then using the false discovery rate (FDR) with the value of *P*_FDR_ ≤ 0.05 as the correction for multiple comparisons. We use ROI binary masks with these clusters and extract the gray-matter volume of ROIs from images in these two groups. As shown in Figure 3, the left part shows the differences of GM probability between AD and NC. The darkness of the negative correlation difference area concentrated in the hippocampus part of the brain, indicating that AD compared with NC in the hippocampus body part with significant atrophy. From right part, we can see significant regions of GM loss between AD and NC in whole brain.

**Figure 4:**
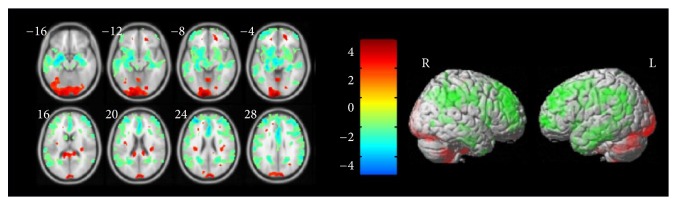
Significant GM difference in MCI relative to NC.

*(2) MCI-NC (Mild Cognitive Impairment Comparing with Normal Controls). *In the experiment of comparing the MCI and NC, we employ an uncorrected threshold (*P*_uncorrected_ ≤ 0.05) then followed by correction for multiple comparisons using the false discovery rate (FDR) (*P*_FDR_ ≤ 0.05) to generate clusters. The significant information of these clusters is shown in Table 3. We select 8 clusters to make the ROI binary mask. Therefore, eight morphometric features were extracted. In Figure 4, we can also find the regions in brain with significant GM differences between MCI and NC. 

**Figure 5:**
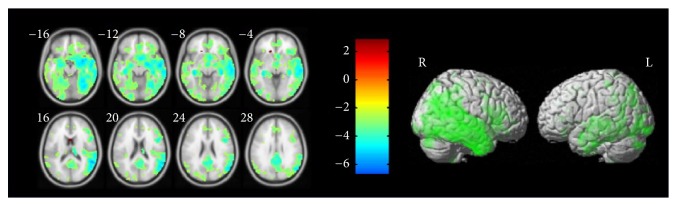
Significant GM difference in AD relative to MCI.

*(3) AD-MCI (Alzheimer's Disease Comparing with Mild Cognitive Impairment).* In the experiment of comparing the MCI and AD, we employ an uncorrected threshold (*P*_uncorrected_ ≤ 0.005), then followed by correction for multiple comparisons using the false discovery rate (FDR) (*P*_FDR_ ≤ 0.05) to generate clusters. The significant information of these clusters is shown in Table 4. We also select 8 clusters. According to Figure 5, we can see the significant GM differences are negative correlation. This is different from the other two experiments.

### 3.3. Assessment of Feature Selection

In order to test the effectiveness of our proposed method SVM-RFE with covariance, several SVM classifiers with different parameters and dataset are used to identify the disease. Moreover, the corresponding accuracy rate for each feature subset was calculated during the process of forward feature selection. The experiment also investigates the effect of *K* on classification accuracy.

*(1) AD-NC (Alzheimer's Disease Comparing with Normal Controls).* When the feature selection is evaluated by the proposed method in this paper, not only are a number of classifiers established to verify the effectiveness of the feature subset, but also the validity of the parameter *K* for the classification effect is obtained and further analyzed. As shown in Table 5, *K* value represents the number of features that need to be selected in the SFS process which have the minimum redundancy with the previous highest ranking feature. The classification result for the training set is the mean accuracy using the optimal feature subset for classification. However, for the test set, the result of classification is optimized and the parameters come from grid search method. When *K* value is 0, the feature selection process ignores the correlation between the features, and it is the same as original SVM-RFE method and the SFS iteration result is a nested feature subset of the sorted feature set. It can be seen from the figure that the average accuracy rates for the training set are lower than other selection processes that do not ignore correlation. When the value of *K* is 1, each time a feature with the lowest redundancy of the highest ranking feature is selected, both the mean accuracy of training set and the optimized accuracy of test set are highest using the selected optimal feature subset. Furthermore, both of best accuracy rates exceed 90%. However, it doesn't mean that the better result can be achieved by setting with the biggest value. When *K* value is 5, the accuracy starts to decline because the method places too much emphasis on the correlation and neglects the importance of the feature itself.

**Figure 6:**
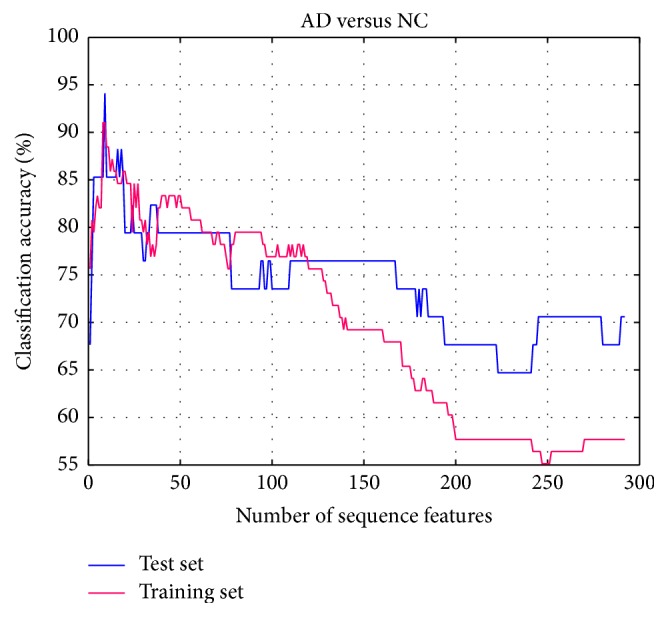
Accuracies obtained by feature selection process (AD-NC).

Based on the experiment results, the parameter *K* is set to 1, and the accuracy rates of the classification AD and NC using optimal feature subset with the method proposed in this paper is shown in Figure 6. It is still the mean accuracy rate for training set, as well as optimized accuracy rate for test set. There are 292-dimensional features that are extracted from the experiment in the feature extracting process. It can be seen from the figure that when the feature subset is initially selected by SFS, both classification accuracies have a significant upward trend. The reason behind this situation is that the feature set is mainly constituted by the most discriminatory features and there is no redundancy in the feature subset. When the feature dimension is 9, both accuracy rates of two-sample set are highest as shown in Figure 6, so we obtain the optimal feature subset at this time. After this, with the growing of number of selected features, the accuracy rates of two-sample set have declined. That is to say, the most discriminative feature combination can be efficiently selected at the beginning, and it also proves the validity of the improved SVM-RFE method proposed in this paper. As shown in the experiment, the robustness of the proposed method can be proved without employing the parameter optimization in the process of feature order and cross validation on training set.

*(2) MCI-NC (Mild Cognitive Impairment Comparing with Normal Controls).* The experimental result of comparing MCI and NC are shown in Table 6, and it is the same as the experiment of comparing AD and NC that *K* value represents the number of features that need to be selected in the SFS process having the minimum redundancy with the previous highest ranking feature. When the value of *K* is 1, each time a feature with the lowest redundancy of the highest ranking feature is selected, both the mean accuracy of training set and the optimized accuracy rate of test set are highest using the selected optimal feature subset. Furthermore, both accuracy rates exceed 95%. We can see that, from comparing the result of *K* = 0 to *K* = 1, both the accuracy rates of training set and test set have sharply increased about 6%. So this can prove the importance of taking the correlation into consideration and the validity of our method.

**Figure 7:**
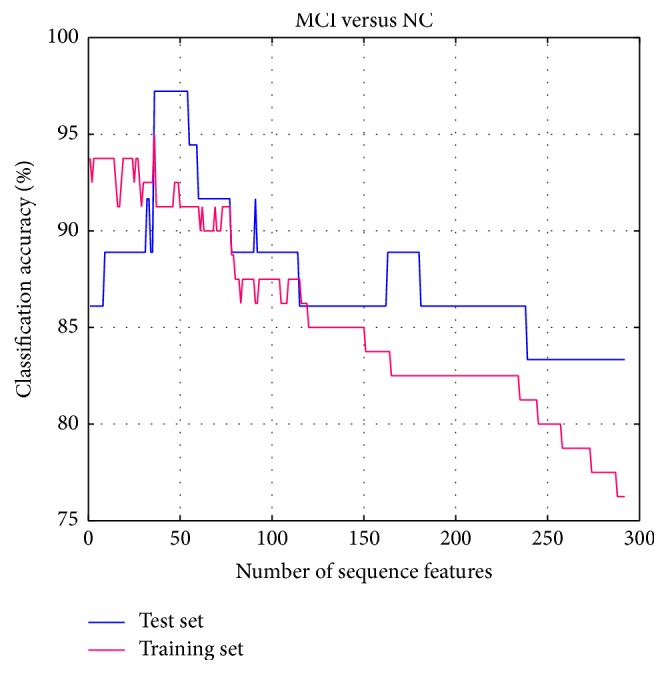
Accuracies obtained by feature selection process (MCI-NC).

For this reason, the parameter *K* is set to 1; the experiment setting and classification result of MCI and NC are the same as the AD and NC. It is also the mean accuracy rate for training set and optimized accuracy rate for test set. When the feature dimension is 36, both accuracy rates of two-sample set are highest as shown in Figure 7, so we obtain the optimal feature subset at this time. After this, with the growing of number of selected features, the accuracy rates of training set have declined. That is to say, the most discriminative feature combination can be efficiently selected at the beginning, and it also proves the validity of the improved SVM-RFE method proposed in this paper.

*(3) AD-MCI (Alzheimer's Disease Comparing with Mild Cognitive Impairment)*. In the experiment of classification of AD and MCI, we also set *K* to represent the number of features that are needed to be selected in the SFS process, which shows the minimum redundancy by employing the previous highest ranking feature. The classification results are shown in Table 7. When *K* = 0, the accuracy rates both on training set and on test set are lower than the others. When *K* = 1, the accuracy rates have increased about 4% which proves the importance of correlation of features. When *K* = 2, both the accuracy rates on two-sample sets achieve the highest about 92%.

**Figure 8:**
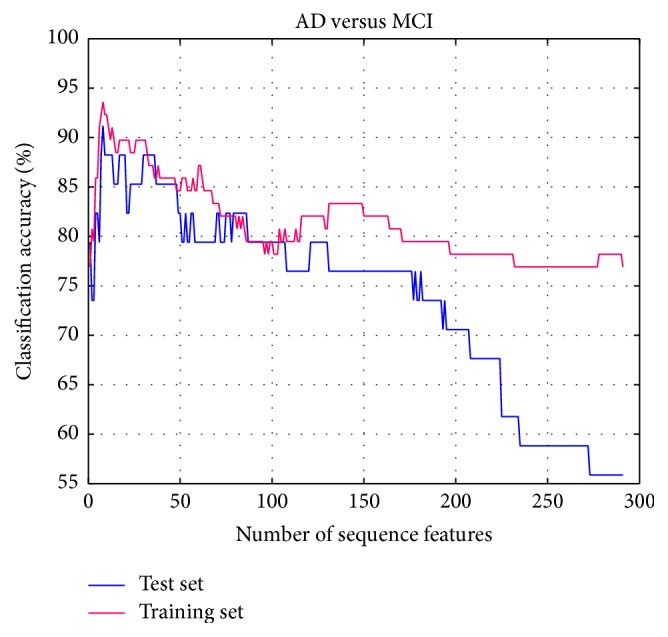
Accuracies obtained by feature selection process (MCI-NC).

Based on the results of our experiment, we set *K* = 2, and the experimental setting and classification result of AD and MCI are the same as the AD and NC. According to Figure 8, both accuracy rates of two-sample sets increase sharply at the beginning of adding the feature into feature subset. When the number of selected features grows to 8, both the accuracy rates of two sample sets are highest. After that, with the increasing of the selected features' numbers, the accuracy starts to decline. It proves the validity of the improved SVM-RFE method proposed in this paper.

*(4) 3-Way Classification (Classification of AD, MCI, and NC).* This is the result of 3-way classification. As can be seen from Table 8, with the increasing of the *K* from 0, the classification accuracy rates (ACC) continuously grow until *K* = 3. When *K* = 3, both of the accuracy rates are the highest and have exceeded 80%. However, with the increasing of *K* from 3, we can see the accuracy starts to decrease; especially when *K* changes from 3 to 4, the accuracy rate decreases about 6% on the test set. So, according to the experimental result, we can find that the proposed method in this paper is still effective and robust in 3-way classification or multiclassification.

**Figure 9:**
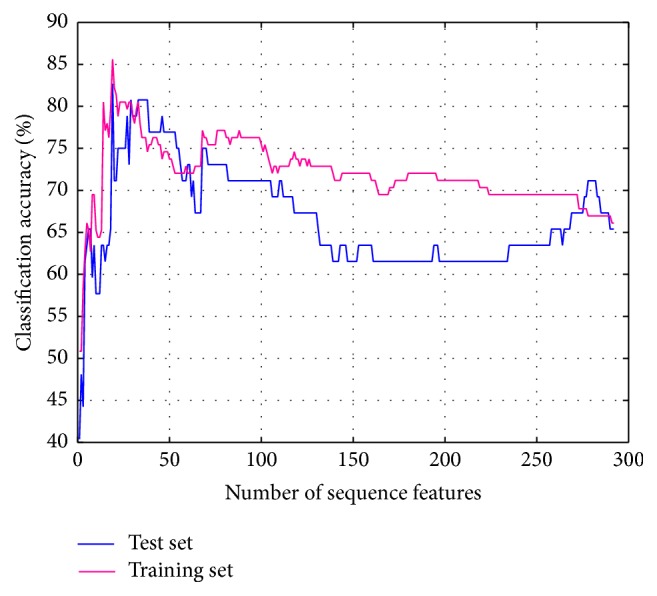
Accuracies obtained by feature selection process (3-way).

For this reason, the parameter is *K* = 3. The classification accuracy for all their feature subsets was plotted in Figure 9. We notice that when the numbers of ranked sequence features and the related features increase from 0 to 19, both accuracy rates on two-sample sets improve sharply. These findings can still prove the effectiveness of our proposed SVM-RFE with covariance method in 3 ways. When the number of selected features in subset is 19, the classification can achieve the best effect, so this feature subset is optimal. When the number of features keeps increasing, the accuracy rates start to decline. So this result demonstrates that our proposed method works well on multiclassification.

In conclusion, the classification performance in the above four experiments has proved the effectiveness of our method. Even though there is no optimal procedure to assign the parameter of SVM1 (Figure 2), our method still shows a good performance which can prove the robustness.

### 3.4. Classification Results

*(1) Performance Measurements.* There are four cases of disease classification results, True Positive (TP), True Negative (TN), False Positive (FP), and False Negative (FN). In our experiments, we considered the following five metrics:

- Accuracy (ACC) = (TP + TN)/(TP + TN + FP + FN).
- Sensitivity (SEN) = TP/(TP + FN).
- Specificity (SPEC) = TN/(TN + FP).
- Positive predictive value (PPV) = TP/(TP + FP).
- Negative predictive value (NPV) = TN/(FN + TN).

The accuracy (ACC) is the most direct metric for comparison between methods. Sensitivity (SEN), specificity (SPEC), positive predictive value (PPV), and negative predictive value (NPV) describe how well diagnostic tests capture the true presence or absence of the disease. These evaluation indexes together describe the accuracy and error rate of recognition method for image classification and recognition. Among them, the higher the ACC, SEN, and SPEC values, the lower the error rate of the recognition method, and PPV and NPV represent the prevalence of the disease in the sample.

*(2) Performance of Feature Combination*. In order to test the effectiveness of the combination of texture features and morphometric features, we adapt the texture features [14] and morphometric features [13] separately to carry out the experiment and then to compare the this accuracy with the accuracy of feature combination method. In the first part of the experiment, the standard SVM with RBF kernel is adopted for classification. The classification performances for the compared methods are summarized in Table 9. As shown in the table, the classification accuracy of feature fusion is obviously improved compared with that of individual features, and there are different degrees of promotion in other indexes, and in particular the specificity index is more obvious. On the other hand, the classification accuracy based on the texture features is almost the same as morphometric features in Alzheimer's disease diagnosis but much better in the mild cognitive impairment diagnosis. (1) AD-NC: the accuracy is 85.71%, showing an increment of at least 6.25%; (2) MCI-NC: the accuracy is 86.11%, showing an increment of at least 2.78%; (3) AD-MCI: the accuracy is 79.44%, showing an increment of at least 2.97%; (4) 3-way classification: the accuracy is 75%, showing an increment of at least 1.92%.

*(3) Performance of the Proposed Method*. In second experiment, in order to test the effectiveness of our proposed method, we compared our method with three other methods as follows: (a) the method without feature selection, (b) PCA [24], and (c) multikernel SVM (Gaussian kernel and polynomial kernel) [32]. To be more specific, in each experiment, there are 220 GLCM features, 64 Gabor features, and 8 morphometric features adapted, such as in feature selection and multikernel SVM. Except for the last method, standard SVM with RBF kernel was adapted for classification. We adapt PCA with 99% cumulate contribution rate and assign equal weight to each kernel. From the result shown in Table 10, we can see that our proposed method performed better than other methods. (1) For classifying AD and NC, our method achieves a classification accuracy of 92.86%, with a sensitivity of 87.04%, a specificity of 98.28%, positive predictive value of 97.28%, and negative predictive value of 89.06%. (2) For classifying MCI and NC, our method achieves a classification accuracy of 97.22%, with a sensitivity of 95.23%, a specificity of 100%, positive predictive value of 100%, and negative predictive value of 93.75%. (3) For classifying AD from MCI, our method achieves a classification accuracy of 91.18%, with a sensitivity of 100%, a specificity of 83.33%, positive predictive value of 84.21%, and negative predictive value of 100%. (4) For 3-way classification, we just have calculated the accuracy rates, and our method achieves a classification accuracy of 85.59%.

According to the experimental result presented above, the proposed method achieves better results than the current two mainstream AD detection methods [13, 14] on the same database ADNI. Through the improved method of feature selection, the classification results have also been promoted again. The detection accuracy of AD is promoted to 92.86% from 79.46% [13] and 78.57% [14], the detection accuracy of MCI is promoted to 97.22% from 63.88% [13] and 83.33% [14], and the detection accuracy of 3-way classification (AD-MCI-NC) is promoted to 85.59% from 63.46% [13] and 73.08% [14]. Furthermore, the other performance measurements (SEN, SPEC, PPV, and NPV) have also been promoted in different degrees. Moreover, a total of 292 features are obtained, and the whole running time of our experiment is just close to 20 seconds. So the proposed method can effectively help doctors to identify the AD patients and the MCI patients without obvious symptoms.

## 4. Conclusion and Future Work

In this paper, a novel feature fusion method is proposed to improve the classification accuracy of AD, MCI, and NC. Firstly, we preprocessed the structural MR images of the subjects and then extracted the morphometric features and texture features. By combining these two kinds of features linearly and then using the feature set to perform classification experiments, we find that the combination of morphometric features and texture features is better than both of them when they were used separately. Based on this, a new feature selection algorithm is proposed which is an improvement of SVM-RFE. By combining SVM-RFE and covariance, the optimal feature subset can be yielded after feature selection process. Finally, we perform several comparison experiments on the public ADNI database using the optimal subset, and then the experimental results were presented and analyzed, which demonstrated the effectiveness of the proposed method in improving classification performance.

The proposed method in this paper effectively promotes the detection accuracy of AD and MCI, but our method still has drawbacks. The next step in our future work is to improve the method from the following aspects: firstly, we try to optimize the obtaining process of parameter *K*. Secondly, we plan to cooperate with the hospital to obtain some real case data, and then we will improve the proposed method and make it more suitable for actual needs of the medical field. Furthermore, in order to improve the effectiveness of the proposed method, the dataset can be extended from the following aspects: (1) increasing the longitudinal dataset to extract effective image identification information at various stages of disease progression for better using in image identification; (2) increasing the multimodal dataset, different imaging techniques that can obtain different characteristic information of the brain which can be integrated to enhance the ability of image recognition.

## Figures and Tables

**Table 1:** Basic information of the subjects.

	AD (*N*^1^ = 54)	MCI (*N*^2^ = 58)	NC (*N* = 58)
Gender (male/female)	22/32	32/26	30/28
Age (mean ± SD^2^)	75.7 ± 7.1	74.8 ± 4.8	75.2 ± 5.6
MMSE (mean ± SD)	22.8 ± 2.3	25.3 ± 1.5	29.1 ± 1.0

(1) *N*^1^, *N*^2^, *N*, number of subjects; (2) SD, Standard Deviation; (3) MMSE, Mini Mental State Examination.

**Table 2:** Information of the significant clusters (AD-NC).

Cluster	Number of voxels	Peak MNI coordinates (*x*, *y*, *z*)	Peak MNI coordinate region
Cluster 1	330608	−30 −10.5 −19.5	Hippocampus
Cluster 2	1565	1.5 −99 −7.5	Calcarine L
Cluster 3	2315	−16.5 −84 −34.5	Cerebelum Crus2 L
Cluster 4	105	0 −40.5 −54	Medulla
Cluster 5	890	1.5 −18 −30	Pons
Cluster 6	2622	40.5 −87 −34.5	Cerebelum Crus1 R
Cluster 7	61	4.5 −94.5 28.5	Occipital Lobe
Cluster 8	1354	21 −25.5 73.5	Frontal Lobe

**Table 3:** Information of the significant clusters (MCI-NC).

Cluster	Number of voxels	Peak MNI coordinates (*x*, *y*, *z*)	Peak MNI coordinate region
Cluster 1	1999	48 −51 −46.5	Cerebellar Tonsil
Cluster 2	9712	−10.5 −103.5 −9	Lingual Gyrus
Cluster 3	173	−13.5 −45 −40.5	Cerebelum_9_L
Cluster 4	107	10.5 −61.5 −39	Uvula
Cluster 5	25	3 −45 −34.5	Vermis_10
Cluster 6	232	18 51 −3	Frontal Lobe
Cluster 7	52	−19.5 42 −3	Anterior Cingulate
Cluster 8	370	−1.5 −63 −4.5	Culmen of Vermis

**Table 4:** Information of the significant clusters (AD-MCI).

Cluster	Number of voxels	Peak MNI coordinates (*x*, *y*, *z*)	Peak MNI coordinate region
Cluster 1	24	1.5 −15 −25.5	Pons
Cluster 2	206	−15 25.5 −4.5	Frontal Lobe
Cluster 3	58	37.5 −43.5 6	Temporal Lobe
Cluster 4	72	12 25.5 10.5	Sub-Lobar
Cluster 5	42	27 19.5 18	Sub-Gyral
Cluster 6	25	30 −7.5 27	Extranuclear
Cluster 7	69	18 −31.5 28.5	Cingulate Gyrus
Cluster 8	43	−12 −25.5 16.5	Pulvinar

**Table 5:** Effect of the number of the related features (AD-NC).

ACC (%)	*K* = 0	*K* = 1	*K* = 2	*K* = 3	*K* = 4	*K* = 5
Test set	88.2	**94.2**	91.0	88.2	88.2	85.1
Training set	82.1	**91.3**	88.4	86.7	97.3	86.7

**Table 6:** Effect of the number of the related features (MCI-NC).

ACC (%)	*K* = 0	*K* = 1	*K* = 2	*K* = 3	*K* = 4	*K* = 5
Test set	88.7	**97.0**	91.7	91.7	94.3	91.7
Training set	90.5	**95.0**	93.7	93.7	93.7	92.5

**Table 7:** Effect of the number of the related features (AD-MCI).

ACC (%)	*K* = 0	*K* = 1	*K* = 2	*K* = 3	*K* = 4	*K* = 5
Test set	85.5	88.2	**91.4**	88.2	88.2	88.2
Training set	87.5	92.2	**93.6**	92.2	91	91

**Table 8:** Effect of the number of the related features (3-way).

ACC (%)	*K* = 0	*K* = 1	*K* = 2	*K* = 3	*K* = 4	*K* = 5
Test set	71.1	78.9	80.8	**82.7**	76.0	75.0
Training set	73.7	80.0	81.7	**85.6**	83.0	78.9

**Table 9:** Classification accuracy with different type of features.

Feature type	ACC (%)	SEN (%)	SEPC (%)	PPV (%)	NPV (%)
AD-NC					
Texture feature	78.57	75.93	81.03	78.85	78.33
Morphological feature	79.46	74.07	84.48	81.36	77.78
Feature combination	**85.71**	**79.63 **	**91.38 **	**89.58**	**82.81**

MCI-NC					
Texture feature	83.33	77.78	88.89	87.50	80.00
Morphological feature	63.88	55.56	65.00	66.67	61.90
Feature combination	**86.11**	**77.78**	**94.44 **	**93.33**	**80.95**

AD-MCI					
Texture feature	76.47	94.44	61.11	70.83	91.67
Morphological feature	70.59	66.67	77.78	75	70
Feature combination	**79.44**	**88.89**	**72.22**	**76.19**	**86.67**

3-way					
Texture feature	73.08	X	X	X	X
Morphological feature	63.46	X	X	X	X
Feature combination	**75.00**	**X**	**X**	**X**	**X**

**Table 10:** Classification performance of all comparison methods.

Method	ACC (%)	SEN (%)	SEPC (%)	PPV (%)	NPV (%)
AD-NC					
Without feature selection	85.71	79.63	91.38	89.58	82.81
PCA	86.71	83.33	87.93	85.64	85.26
Multikernel SVM	88.39	85.19	91.38	90.20	86.89
Proposed method	**92.86**	**87.04 **	**98.28**	**97.28**	**89.06**

MCI-NC					
Without feature selection	86.11	77.78	94.44	93.33	80.95
PCA	86.11	85.71	86.67	90.00	81.25
Multikernel SVM	91.67	90.47	93.33	95.00	87.50
Proposed method	**97.22**	**95.23 **	**100**	**100**	**93.75**

AD-MCI					
Without feature selection	79.44	88.89	72.22	76.19	86.67
PCA	73.53	81.25	66.67	68.42	80.00
Multikernel SVM	79.41	87.50	72.22	73.68	86.67
Proposed method	**91.18**	**100 **	**83.33**	**84.21**	**100**

3-way					
Without feature selection	75.00	X	X	X	X
PCA	69.23	X	X	X	X
Multi-kernel SVM	79.41	X	X	X	X
Proposed method	**85.59**	**X**	**X**	**X**	**X**
